# Detecting DSM-5 somatic symptom disorder in general hospitals in China: B-criteria instrument has better accuracy—A secondary analysis

**DOI:** 10.3389/fpsyt.2022.935597

**Published:** 2022-10-20

**Authors:** Jinya Cao, Jing Wei, Kurt Fritzsche, Anne Christin Toussaint, Tao Li, Lan Zhang, Yaoyin Zhang, Hua Chen, Heng Wu, Xiquan Ma, Wentian Li, Jie Ren, Wei Lu, Rainer Leonhart

**Affiliations:** ^1^Department of Psychological Medicine, Peking Union Medical College Hospital, Chinese Academy of Medical Sciences and Peking Union Medical College, Beijing, China; ^2^Center for Mental Health, Department of Psychosomatic Medicine and Psychotherapy, Faculty of Medicine, Medical Centre - University of Freiburg, Freiburg im Breisgau, Germany; ^3^Department of Psychosomatic Medicine and Psychotherapy, University Medical Centre Hamburg-Eppendorf, Hamburg, Germany; ^4^Mental Health Centre, West China Hospital, Sichuan University, Chengdu, China; ^5^Department of Psychosomatic Medicine, Sichuan Provincial People's Hospital, University of Electronic Science and Technology of China, Chengdu, China; ^6^Department of Psychological Medicine, Zhong Shan Hospital, Fudan University, Shanghai, China; ^7^Department of Psychosomatic Medicine, Tongji Hospital, School of Medicine, Tongji University, Shanghai, China; ^8^Department of Psychosomatic Medicine, Dongfang Hospital, School of Medicine, Tongji University, Shanghai, China; ^9^Department of Clinic Psychology, Wuhan Mental Health Centre, Wuhan, China; ^10^Department of Rehabilitation, General Hospital of Jincheng Anthracite Coal Mining Group Co., Ltd., Jincheng, China; ^11^Department of Psychosomatic Medicine, Beijing Hospital of Traditional Chinese Medicine, Capital University, Beijing, China; ^12^Institute of Psychology, University of Freiburg, Freiburg im Breisgau, Germany

**Keywords:** somatic symptom disorder, PHQ-15, SSS-8, SSD-12, WI-8

## Abstract

**Objective:**

This study investigates the diagnostic accuracy of the PHQ-15, SSS-8, SSD-12 and Whitley 8 and their combination in detecting DSM-5 somatic symptom disorder in general hospitals.

**Methods:**

In our former multicenter cross-sectional study enrolling 699 outpatients from different departments in five cities in China, SCID-5 for SSD was administered to diagnose SSD and instruments including PHQ-15, SSS-8, SSD-12 and WI-8 were used to evaluate the SSD A and B criteria. In this secondary analysis study, we investigate which instrument or combination of instrument has best accuracy for detecting SSD in outpatients. Receiver operator curves were created, and area under the curve (AUC) analyses were assessed. The sensitivity and specificity were calculated for the optimal individual cut points.

**Results:**

Data from *n* = 694 patients [38.6% male, mean age: 42.89 years (SD = 14.24)] were analyzed. A total of 33.9% of patients fulfilled the SSD criteria. Diagnostic accuracy was moderate or good for each questionnaire (PHQ-15: AUC = 0.72; 95% CI = 0.68–0.75; SSS-8: AUC = 0.73; 95% CI = 0.69–0.76; SSD-12: AUC = 0.84; 95% CI = 0.81–0.86; WI-8: AUC = 0.81; 95% CI = 0.78–0.84). SSD-12 and WI-8 were significantly better at predicting SSD diagnoses. Combining PHQ-15 or SSS-8 with SSD-12 or WI-8 showed similar diagnostic accuracy to SSD-12 or WI-8 alone (PHQ-15 + SSD-12: AUC = 0.84; 95% CI = 0.81–0.87; PHQ-15 + WI-8: AUC = 0.82; 95% CI = 0.79–0.85; SSS-8 + SSD-12: AUC = 0.84; 95% CI = 0.81–0.87; SSS-8 + WI-8: AUC = 0.82; 95% CI = 0.79–0.84). In the efficiency analysis, both SSD-12 and WI-8 showed good efficiency, SSD-12 slightly more efficient than WI-8; however, within the range of good sensitivity, the PHQ-15 and SSS-8 delivered rather poor specificity. For a priority of sensitivity over specificity, the cutoff points of ≥13 for SSD-12 (sensitivity and specificity = 80 and 72%) and ≥17 for WI-8 (sensitivity and specificity = 80 and 67%) are recommended.

**Conclusions:**

In general hospital settings, SSD-12 or WI-8 alone may be sufficient for detecting somatic symptom disorder, as effective as when combined with the PHQ-15 or SSS-8 for evaluating physical burden.

## Introduction

Somatic symptom disorder (SSD) was introduced in the DSM-5 in 2013 (1). The diagnosis of SSD is made when there are persistent (typically more than 6 months, Criteria C) and clinically significant somatic complaints (Criteria A) that are accompanied by excessive and disproportionate health-related thoughts, feelings, and behaviors regarding these symptoms (B-type criteria). Somatic complaints can be caused by medical diseases (organic) or not caused by them (functional). SSD is meant to substitute somatoform disorder in DSM-IV by avoiding the discussion of whether the somatic symptom can be medically explained or not. Moreover, the content of SSD is now further extended by including patients whose complaints can be explained by medical diseases. There have been doubts that SSD may be overinclusive as it includes medical patients with appropriate psychological reactions; however, it has been found that even in patients with a major medical burden, such as heart disease or arthritis, a diagnosis of SSD is not automatic (2). Only a fraction of such patients with chronic, persistent and distressing somatic complaints can be diagnosed with SSD. Indeed, it is the combination of somatic symptoms and B-type criteria that is associated with worsened quality of life and increased healthcare use (3). We think this diagnostic extension of including medical patients is especially meaningful, as this creates an opportunity to offer help to medical patients suffering from psychological burdens that are related to (and may also influence) medical complaints.

Clinical interviews are always most reliable in making diagnoses; however, they can be very time-consuming. In a real-world situation, in the limited time set of outpatient clinics, there is difficulty felt by both doctors and patients in achieving mutual empathy and understanding, not to mention to accomplish a full and extensive clinical interview. Thus, efficient screening tools of possible somatic symptom disorder can help greatly in clinic.

Well-established tools such as the Patient Health Questionnaire-15 (4) or the Somatic Symptom Scale-8 (5) can assist in assessing the A criteria of distressing somatic symptoms. The Somatic Symptom Disorder—B Criteria Scale (SSD-12) was developed to assess the psychological B criteria of SSD (6) and has been shown to have good validity in detecting SSD (7). A study in a German psychosomatic outpatient population showed that the combination of the PHQ-15 or SSS-8 with the SSD-12 increased the validity of identifying SSD compared with using each instrument alone (8).

In our former study (9), we found a prevalence of 33.6% (236/699) in a Chinese outpatient population and that SSD is associated high physical and psychological burdens and social function impairment. Drawing on the experience of Toussaint et al. (8), here we present a secondary data analysis of our former study (9) to investigate the predictive values of the PHQ-15, SSS-8, SSD-12 and WI-8 used alone or in combination for detecting SSD in Chinese general hospital outpatient clinics.

## Methods

### Study design and subjects

Our former multicenter cross-sectional study was conducted between May 2016 and March 2017 in the outpatient clinics of the neurology, gastroenterology, Traditional Chinese Medicine [TCM] and psychosomatic medicine departments of nine tertiary hospitals in Beijing, Jincheng, Shanghai, Wuhan, and Chengdu (located in the north, north-central, east, central, and southwest regions of China, respectively).

For inclusion in the study, the participants were required to be at least 18 years of age, to be visiting for treatment (i.e., not only picking up a prescription), to have adequate reading and writing skills and to have signed a written consent form. Exclusion criteria included the presence of language barriers, limited reading skills, cognitive impairment, acute psychosis or suicidal tendency.

The diagnosis of SSD was made by diagnostic SCID-5 interviews by trained clinical researchers blind to the screening scale results.

A detailed description of the procedure can be found in our previously published article (9).

### Instruments

The PHQ-15 and SSS-8 for the A criteria and the SSD-12 and WI-8 for the B criteria were administered:

Somatic Symptom Severity Scale of the Patient-Health-Questionnaire (PHQ-15):

The PHQ-15 assesses 15 somatic symptoms, such as fatigue, pain, and gastrointestinal, musculoskeletal, and cardiopulmonary symptoms within the last 4 weeks. Each symptom is scored from 0 (“not bothered at all”) to 2 (“bothered a lot”). Sum scores range from 0 to 30 and indicate the self-rated symptom burden (0–4 no to minimal; 5–9 low; 10–14 medium; 15–30 high). The Chinese version of the PHQ-15 exhibits satisfactory reliability (10) and validity (11).

Somatic Symptom Scale-8:

The SSS-8 is an abbreviated version of the PHQ-15, which was developed within DSM-5 field trials (12). A five-point response option (0–4) for each item and a 7-day time frame were used. The cutoff scores indicated whether a patient suffered from minimal (0–3 points), low (4–7), medium (8–11), high (12–15), or very high (16-32) somatic symptom burden. Previous studies demonstrated good item characteristics and excellent reliability, sound factor structures, and significant associations with related constructs such as depression, anxiety, quality of life, and health care use (5). These results have not been validated in China. In this sample, we estimated a Cronbach's alpha of 0.783.

Somatic Symptom Disorder—B Criteria Scale 12:

The Somatic Symptom Disorder—B Criteria Scale 12 (SSD-12) is composed of 12 items. Each of the three psychological sub-criteria is measured by four items with all item scores ranging between 0 and 4. The external and internal validity of this method have been established (6, 13). A cutoff point of 16 or 17 for SSD-12 has been found in Chinese studies for detecting SSD (7, 14).

Whiteley-8:

The Whiteley-8 test measures health-related anxiety in the previous 4 weeks. It has 8 items on a five-point Likert scale. In our study, each item score ranged between 1 and 5. The original well-validated 7-item scale WI-7 (15) was extended by one additional item: “Recurring thoughts about having a disease that is difficult to be rid of?” This item of rumination seemed to capture one core characteristic of health anxiety (16). The WI-8 was first used in the Danish study of functional disorders (17). The Chinese version of the WI-7 exhibited satisfactory reliability and internal validity in a general population sample (18, 19). The WI-8 has also been validated in China (20). In this sample, we estimated a Cronbach's alpha of 0.937.

### Statistical procedures

The study center at Peking Union Medical College Hospital (PUMCH) stored all the data, regularly monitored all project sites and analyzed the data.

Analyses were carried out using the Statistical Package for the Social Sciences version 25.0 (SPSS Inc., Chicago, IL, USA) and MedCalc Version 20.

## Results

### Sample characteristics

In total, 1,269 participants were approached, and the response rate was 55.08%. A total of 697 participants were presented in our former study, as 3 of them had missed questionnaire data, a total of 694 participants who completed both the interview and the questionnaires of this study are presented in this study. Two hundred twenty-four participants came from the gastroenterology/neurology department, 239 from the psychosomatic medicine department, and 231 from the TCM department.

Among the 694 participants, 235 (33.9%) were diagnosed with SSD according to the SCID-5 interview. The average age of the participants was 42.89 years (SD = 14.24). Among them, 38.6% were male. There were no differences in age, sex, health insurance status, residence status, marital status, family income, occupation status, education, physical disease diagnosis or physical disease severity between the SSD group and the non-SSD group (Table 1).

**Table 1:** Baseline data of the study sample (*N* = 694).

		**Total (*****N*** = **694)**		**With SSD (*****N*** = **235)**		**Without SSD (*****N*** = **459)**				
		**Mean**	**SD**	**Mean**	**SD**	**Mean**	**SD**			***p***
Age		42.89	14.24	42.96	14.07	42.86	14.35			0.932
		***N***	%	***N***	%	***N***	%	**Chi** ^**2**^	***df***	***p***
Gender	Female	426	61.4	142	60.4	284	61.90	0.137	1	0.711
Health insurance	Yes	597	86.9	199	85.34	398	87.70	0.689	1	0.406
	No	90	13.00	34	14.50	56	12.30			
Residence	City	571	82.40	186	79.50	385	83.90	2.06	1	0.151
	Country	122	17.60	48	20.50	74	16.10			
Marital status	Single	129	18.60	45	19.10	84	18.30	7.38	5	0.194
	Married	503	72.50	162	68.90	341	74.30			
	Seperated	4	0.60	3	1.30	1	0.20			
	Divorced	38	5.50	17	7.20	21	4.60			
	Widowed	12	1.70	6	2.60	6	1.30			
	Others	8	1.20	2	0.90	6	1.30			
Family income	Low (under 4,000 RMB^a^)	233	33.70	89	38.00	144	31.50	3.40	2	0.183
	Middle (4,000–8,000 RMB)	242	35.00	80	34.20	162	35.40			
	High (above 8,000 RMB)	216	31.30	65	27.80	151	33.00			
Occupation	Employed	341	49.10	105	44.70	236	51.40	6.78	5	0.238
	Unemployed	84	12.10	38	16.30	46	10.20			
	Retire	149	21.50	51	21.70	98	21.40			
	Housewife	44	6.30	16	6.80	28	6.10			
	Student	39	5.60	14	6.00	25	5.40			
	Others	37	5.30	11	4.70	26	5.70			
Education	Primary school	45	6.50	18	7.70	27	5.90	4.15	3	0.246
	Middle school	135	19.50	54	23.00	81	17.60			
	Higher school	179	25.80	58	24.70	121	26.40			
	University or higher	335	48.30	105	44.70	230	50.10			
Physical disease	No	417	60.1	143	60.9	274	59.7	0.087	1	0.769
	Yes	277	39.9	92	39.1	185	40.3			
Physical disease severity grade	0	417	60.1	143	60.9	274	59.7	2.075	3	0.557
	1	116	16.7	44	18.7	72	15.7			
	2	128	18.4	38	16.2	90	19.6			
	3	33	4.8	10	4.3	23	5.0			
Scale scores	Somatic symptom severity (PHQ-15) range = 0–30	9.33	5.38	12.00	5.53	7.96	4.77			< 0.001
	Somatic symptom severity (SSS-8) range = 0–32	8.70	6.08	11.98	6.51	7.02	5.0			< 0.001
	Psychological symptom severity (SSD-12) range = 0–48	13.98	12.24	23.58	11.45	9.07	9.38			< 0.001
	Whiteley 8 (WI-8) range = 8–40	18.23	8.34	24.41	8.44	15.06	6.26			< 0.001

Percentages are normally column percentages. ^*a*^RMB: The renminbi is the currency of the People's Republic of China; 1,000 RMB is equivalent to ~125 Euro.

The PHQ-15, SSS-8, SSD-12 and WI-8 scores were significantly different between the SSD group and the non-SSD group (Table 1). But these scores showed similar distribution between participants with physical disease and without physical disease, and these scores showed no correlation with physical disease severity (Supplementary Table S0).

### Descriptive item reliability

The SSD-12 showed the highest reliability in this sample (α = 0.937). Cronbach's α values for the PHQ-15, SSS-8, SSD-12 and WI-8 assessments in this sample were 0.809, 0.783, 0.954, and 0.937, respectively. These predictors were moderately to very highly correlated (Supplementary Table S1).

### Correlation of predictors

A Pearson correlation analysis showed that SSD-12 and WI-8 were very highly correlated, PHQ-15 and SSS-8 were highly correlated, PHQ-15 and SSD-12/WI-8 were moderately correlated, and SSS-8 and SSD-12/WI-8 were highly correlated (Table 2).

**Table 2:** Stepwise logistic regression analysis evaluating the PHQ-15/SSS-8 and SSD-12/WI-8 as predictors for SSD diagnosis (*n* = 694).

**Variables**	**B**	**SE**	***p***	**OR**	**95% CI**
**PHQ-15 and SSD-12**					
Step 1					
PHQ-15	0.151	0.017	< 0.001	1.163	1.124–1.203
Constant	−2.151	0.196	< 0.001	0.116	
Step 2					
PHQ-15	0.052	0.020	0.011	1.053	1.012–1.096
SSD-12	0.106	0.010	< 0.001	1.112	1.091–1.134
Constant	−2.851	0.236	< 0.001	0.058	
**SSS-8 and WI-8**					
Step 1					
SS-8	0.144	0.015	< 0.001	1.155	1.121–1.190
Constant	−2.004	0.171	< 0.001	0.135	
Step 2					
SSS-8	0.047	0.019	0.013	1.048	1.010–1.087
WI-8	0.139	0.015	< 0.001	1.150	1.117–1.184
Constant	−3.781	0.278	< 0.001	0.023	
**SSD-12 and PHQ-15**					
Step 1					
SSD-12	0.117	0.009	< 0.001	1.124	1.104–1.144
Constant	−2.491	0.179	< 0.001	0.083	
Step 2					
SSD-12	0.106	0.010	< 0.001	1.112	1.091–1.134
PHQ-15	0.052	0.020	0.011	1.053	1.012–1.096
Constant	−2.851	0.236	< 0.001	0.058	
**WI-8 and SSS-8**					
Step 1					
WI-8	0.158	0.013	< 0.001	1.172	1.142–1.202
Constant	−3.708	0.274	< 0.001	0.025	
Step 2					
WI-8	0.139	0.015	< 0.001	1.150	1.117–1.184
SSS-8	0.047	0.019	0.013	1.048	1.010–1.087
Constant	−3.781	0.278	< 0.001	0.023	

For the simplicity of data representation, only the PHQ-15 and SSD-12 combination and SSS-8 and WI-8 combination are listed here.

Model summary statistics:

PHQ-15 and SSD-12.

Step 1: step: χ^2^ (1) = 89.67, p < 0.001; model: −2 log likelihood =798.81; Cox and Snell R^2^ = 0.12; Nagelkerke R^2^ = 0.17. Step 2: step: χ^2^ (1) = 239.70, p < 0.001; model: −2 log likelihood = 648.78; Cox and Snell R^2^ = 0.29; Nagelkerke R^2^ = 0.40.

SSS-8 and WI-8.

Step 1: step: χ^2^ (1) = 104.91, p < 0.001; model: −2 log likelihood = 783.56; Cox and Snell R2 = 0.14; Nagelkerke R^2^ = 0.19. Step 2: step: χ^2^ (1) = 212.09, p < 0.001; model: −2 log likelihood = 676.39; Cox and Snell R^2^ = 0.26; Nagelkerke R^2^ = 0.37.

SSD-12 and PHQ-15.

Step 1: step: χ^2^ (1) = 233.21, p < 0.001; model: −2 log likelihood = 655.27; Cox and Snell R^2^ = 0.29; Nagelkerke R^2^ = 0.40. Step 2: step: χ^2^ (1) = 239.70, p < 0.001; model: −2 log likelihood = 648.78; Cox and Snell R^2^ = 0.29; Nagelkerke R^2^ = 0.40.

WI-8 and SSS-8.

Step 1: χ^2^ (1) = 205.89, p < 0.001; model: −2 log likelihood = 682.59; Cox and Snell R^2^ = 0.26; Nagelkerke R^2^ = 0.36. Step 2: step: χ^2^ (1) = 212.09, p < 0.001; model: −2 log likelihood = 676.39; Cox and Snell R^2^ = 0.26; Nagelkerke R^2^ = 0.37.

B, unstandardized regression coefficient; SE, standard error; OR, odds ratio; CI, confidence interval.

### Combination of screening instruments

The combination of the PHQ-15/SSS-8 with the SSD-12/WI-8 in the regression analysis showed significantly more variance in predicting SSD than the PHQ-15/SSS-8 alone, while the combinations showed no improvement over SSD-12/WI-8 alone (as shown by the *R*^2^ differences listed in Table 2).

### ROC analysis

From ROC curve analysis with MedCalc, the cutoff point for PHQ-15 was found to be ≥8, with a sensitivity of 80%, specificity of 52%, and Youden Index of 0.32 (as there is an item related to menstruation, when considered separately, the cutoff point of PHQ-15 with highest Youden index should be 8 for women and 7 for men); the cutoff point for SSS-8 was ≥9, with a sensitivity of 67%, specificity of 68%, and Youden Index of 0.35; the cutoff point for SSD-12 was ≥16, with a sensitivity of 76%, specificity of 80%, and Youden Index 0.56; the cutoff point for WI-8 was ≥19, with a sensitivity of 73%, specificity of 76%, and Youden Index of 0.49.

**Figure 1:**
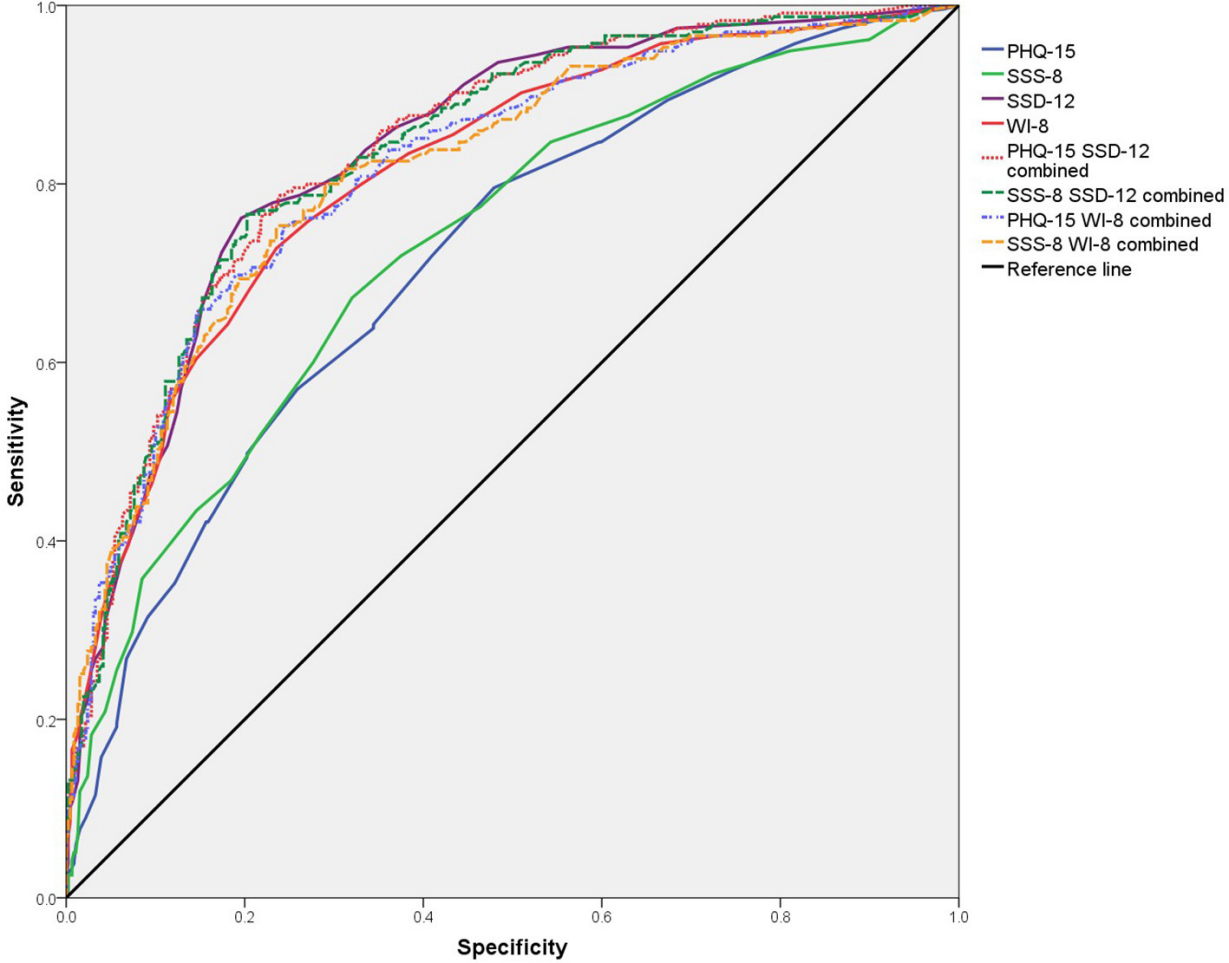
ROC curves of PHQ-15, SSS-8, SSD-12, WI-8, and their combinations.

The PHQ-15 (AUC = 0.715) and SSS-8 (AUC = 0.729) showed moderate diagnostic accuracy, while SSD-12 (AUC = 0.837) and WI-8 (AUC = 0.813) demonstrated good diagnostic accuracy. The differences between PHQ-15 or SSS-8 and SSD-12 or WI-8 were statistically significant (Supplementary Tables S2, S3, Figure 1).

The combination of the A and B criteria showed no significant improvement compared to the B criteria alone (PHQ-15 + SSD-12: AUC = 0.838; PHQ-15 + WI-8: AUC = 0.818; SSS-8 +SSD-12: AUC = 0.836; SSS-8 + WI-8: AUC = 0.816; Supplementary Tables S2, S3, Figure 1).

### Diagnostic accuracy

However, since the potential harm of a false positive diagnosis and a false negative diagnosis is not equal in the case of SSD, the Youden Index may not be the best consideration when choosing cutoff points. A sensitivity priority should be considered, since a missed diagnosis would cause more harm than a false positive diagnosis, which would cause more clinical evaluation efforts but no damage. A diagnostic accuracy analysis according to Toussaint et al. (8) was performed to find a more sensitive cutoff point for each instrument (Supplementary Table S4). Only relevant ranges are shown.

Cutoff points of ≥13 for SSD-12 (sensitivity and specificity = 80 and 72%) and ≥17 for WI-8 (sensitivity and specificity = 80 and 67%) could be used.

Since previous studies reported severity thresholds of ≥10 (medium somatic symptom burden) and ≥15 (high somatic symptom burden) for both the PHQ-15 and the SSS-8 (21), and the corresponding thresholds for the SSD-12 can be determined at ≥20 and ≥25 (13), the cutoff points obtained with these combinations are also reported in Table 3. As there are no existing cutoff points for the WI-8 from other studies, no similar analysis was performed with the WI-8.

**Table 3:** Combination of relevant cutoff points of PHQ-15 and SSD-12, and SSS-8 and SSD-12 (*n* = 694).

**PHQ-15 and SSD-12**	**Optimal cutoff points determined in the current sample** **PHQ-15 ≥8 and SSD-12 ≥13**	**Pragmatic cutoff points based on established severity scores (medium severity)** **PHQ-15 ≥10 and SSD-12 ≥20**	**Pragmatic cutoff points based on established severity scores (high severity)** **PHQ-15 ≥15 and SSD-12 ≥25**
Sensitivity	0.68	0.48	0.21
Specificity	0.80	0.92	0.98
NPV	0.64	0.76	0.82
PPV	0.83	0.77	0.71
Efficiency	0.76	0.77	0.72
SSS-8 and SSD-12	Optimal cutoff points determined in the current sample SSS-8 ≥ 9 and SSD-12 ≥ 13	Pragmatic cutoff points based on established severity scores (medium severity) SSS-8 ≥ 10 and SSD-12 ≥ 20	Pragmatic cutoff points based on established severity scores (high severity) SSS-8 ≥ 15 and SSD-12 ≥ 25
Sensitivity	0.62	0.50	0.25
Specificity	0.85	0.92	0.97
NPV	0.69	0.75	0.78
PPV	0.82	0.78	0.71
Efficiency	0.78	0.77	0.72

The application of higher severity cutoff points as determined by previous studies did not increase the efficiency but did decrease the sensitivity to an insufficient level.

## Discussion

The present study evaluates and compares the diagnostic accuracy of the PHQ-15, SSS-8, SSD-12, and WI-8 and their combination for detecting DSM-5 somatic symptom disorder within a sample of general hospital outpatients. At their cutoff points from ROC analysis, SSS-8 (≥7) showed a relatively poor sensitivity and specificity; PHQ-15 (≥6) showed a high sensitivity, but a low specificity; however, SSD-12 (≥14) and WI-8 (WI ≥ 17) both showed good sensitivities and specificities. Combining the PHQ-15 or SSS-8 (to assess the A criteria) with the SSD-12 or WI-8 (to assess the B criteria) did not further increase the AUC compared to the use of the SSD-12 or WI-8 alone.

Previous studies investigating the use of PHQ-15, SSS-8, WI-7 and SSD-12 in detecting functional somatic symptoms or somatic symptom disorders in psychiatric populations or the general population have generally found good validity for these instruments (5, 8, 10, 11, 18, 19). The combination of an A criteria instrument (PHQ-15 or SSS-8) and a B criteria instrument (SSD-12) slightly improved the diagnostic accuracy (8).

In the study by Liao et al. (22) in psychiatric outpatients and healthy controls, the PHQ-15 scores of SSD patients and non-SSD patients were 10.04 (±6.03, *n* = 200) and 5.69 (±4.72, *n* = 271), respectively, and the cutoff point determined for the PHQ-15 was 4/5. The study by Toussaint et al. (8) was performed with psychiatric outpatients; the PHQ-15 scores of SSD patients and non-SSD patients were 14.6 (±5.0, *n* = 209) and 11.1 (±4.7, *n* = 163), respectively, and the cutoff point determined for the PHQ-15 was ≥9. Physical symptoms such as pain, fatigue, heart palpitation, shortness of breath and gastroenterological symptoms are common and distressful in patients with depression and anxiety (23, 24). Depression and anxiety can also increase somatic symptom severity in organic disease patients (25). It is expected that the PHQ-15 score should be higher in the psychiatric outpatient group. The cutoff point for the PHQ-15 determined in our study was ≥8, in between those found in the previous two studies.

SSS-8 is an abbreviated version of the PHQ-15. In past studies, it has been demonstrated to have a similar efficiency as the PHQ-15 in screening for bodily symptoms (21). This is the same case in our study: SSS-8 had similar AUC and efficiency as PHQ-15. Within a good range of sensitivity, PHQ-15 and SSS-8 would both show poor specificity. This may be because in general hospitals, “genuine” bodily symptoms are relatively more likely and more frequent than in general populations or psychiatric hospitals; therefore, the A criteria would have a low specificity.

SSD-12 and WI-8 both showed good efficiency in our study, and the SSD-12 was slightly better than WI-8. This agrees with the core concept of SSD: that it is the psychological symptoms associated with the physical burdens, not the physical burdens themselves, that define SSD (2). In our sample, the finding that there is no difference of SSD diagnosis or instruments scores between different groups of physical disease status, also supports this idea that physical conditions themselves does not necessarily cause a higher chance of SSD or higher SSD severity. What defines SSD psychopathology is the psycho-behavior reaction to a somatic symptom, whether the symptom is organic or functional, whether the symptom is severe or not.

The cutoff point determined for SSD-12 (cutoff point ≥16) from the ROC analysis in our study was much lower than that found in the study by Toussaint et al. (8) (SSD-12 cutoff point ≥26). This difference could also be explained by participant selection differences. As the SSD-12 total sum-score was significantly associated with general anxiety and depressive symptoms (6, 26), it is expected that in a sample of psychiatric patients, the cutoff point for the SSD-12 would be higher.

In summary, the results from our study suggest that in general hospital outpatient settings, it's hard to find a good balance of sensitivity and specificity for PHQ-15 and SSS-8. So these instruments may be best used to evaluate SSD severity, but not as screening tools. In contrast, SSD-12 and WI-8 show good diagnostic accuracy. One B-criteria instrument seems to be sufficient by itself, with no further need or benefit of combining with one A-criteria instrument. When sensitivity is prioritized over specificity, the recommended cutoff points are ≥13 for SSD-12 and ≥17 for WI-8.

One limitation of our study is that only gastroenterological and neurological departments were chosen for biomedical departments, and approximately equal numbers of participants were selected from the biomedical, TCM and psychological departments, which may not represent the ratio of help-seekers to different departments in general hospitals. Different departments may have their own characteristic profiles of SSD presentation. Further detailed investigations in different clinical specialties from the perspective of consultation-liaison services may be warranted. Also, as our study was conducted in tertiary hospitals, the result may not be generalizable to primary care where patients with a less severe symptomatology present themselves.

## Conclusion

In general hospital settings, SSD-12 or WI-8 alone may be sufficient for detecting somatic symptom disorder, as effective as when combined with PHQ-15 or SSS-8, while PHQ-15 and SSS-8 show a relatively poor diagnostic accuracy.

## Data availability statement

The original contributions presented in the study are included in the article/Supplementary material, further inquiries can be directed to the corresponding author.

## Ethics statement

The studies involving human participants were reviewed and approved by the Ethics Committees of Peking Union Medical College Hospital (PUMCH) and the University Medical Center, Freiburg, Germany (Protocol Number: S-K276). The patients/participants provided their written informed consent to participate in this study.

## Author contributions

JW and KF designed this study. JW, TL, WLu, LZ, YZ, HW, XM, HC, WLi, and JR coordinated the study. JC made the drafting and statistic analysis. JW, KF, RL, and AT made critical reviews and improvement of the draft. All authors contributed to the article and approved the submitted version.

## Funding

JC and JW is supported by funds of the Ministry of Science and Technology, People's Republic of China (2021ZD0202001) and Capital's Funds for Health Improvement and Research (CFH 2022-2-4012) and National High Level Hospital Clinical Research Funding.

## Conflict of interest

Author JR is employed by General Hospital of Jincheng Anthracite Coal Mining Group Co., Ltd. The remaining authors declare that the research was conducted in the absence of any commercial or financial relationships that could be construed as a potential conflict of interest.

## Publisher's note

All claims expressed in this article are solely those of the authors and do not necessarily represent those of their affiliated organizations, or those of the publisher, the editors and the reviewers. Any product that may be evaluated in this article, or claim that may be made by its manufacturer, is not guaranteed or endorsed by the publisher.
