# Supplementary material for: Detecting DSM-5 somatic symptom disorder in general hospitals in China: B-criteria instrument has better accuracy—A secondary analysis
Source: Front Psychiatry. 2022 Oct 20;13:935597. doi: 10.3389/fpsyt.2022.935597 (PMC9634742; doi:10.3389/fpsyt.2022.935597)
Supplement: Supplementary file 1 [file Data_Sheet_1.docx]

**Supplementary Tables**

**Table S0. Instrument scores in participants with and without physical disease, and instrument score correlations with physical disease severity**

|  |  | **Instrument scores in participants** | | | | | |  | **Correlation with physical disease severity(0-3)** | |
| --- | --- | --- | --- | --- | --- | --- | --- | --- | --- | --- |
|  |  | **Total**  **(N=694)** |  | **With physical disease**  **(N=277)** | | **Without physical disease**  **(N=417)** | |  |  |  |
|  |  | **mean** | **SD** | **mean** | **SD** | **mean** | **SD** | p | **rho** | **p** |
| **Scale scores** | Somatic symptom severity (PHQ-15) range = 0–30 | 9.33 | 5.38 | 9.43 | 5.25 | 9.27 | 5.48 | .485 | 0.027 | .0.471 |
|  | Somatic symptom severity (SSS-8) range = 0–32 | 8.70 | 6.08 | 8.73 | 5.99 | 8.68 | 6.16 | .414 | 0.016 | 0.668 |
|  | Psychological symptom severity (SSD-12) range = 0–48 | 13.98 | 12.24 | 13.92 | 12.19 | 14.03 | 12.28 | .649 | .0.003 | 0.935 |
|  | Whiteley 8(WI-8) range=8-40 | 18.23 | 8.34 | 18.60 | 8.27 | 17.99 | 8.39 | .768 | 0.047 | .0.218 |

**Table S1. Pearson Correlation Coefficient between predictors (n=694)**

|  | **PHQ-15** | **SSS-8** | **SSD-12** | **WI-8** |
| --- | --- | --- | --- | --- |
| **PHQ-15** | 1 | . 738** | .518** | .550** |
| **SSS-8** | .738** | 1 | .606** | .608** |
| **SSD-12** | .518** | .606** | 1 | .824** |
| **WI-8** | .550** | .608** | .824** | 1 |

Notes: A Pearson Correlation Coefficient of 0.8-1.0 indicates very highly correlated, of 0.6-0.8 indicated highly corelated, of 0.4-0.6 indicated moderately correlated. p<0.001**

**Table S2. ROC curves of PHQ-15, SSS-8, SSD-12 and WI-8 and A and B Criterion combinations**

| Instrument | AUC | SE ^a^ | 95% CI ^b^ |
| --- | --- | --- | --- |
| PHQ-15 | 0.715 | 0.0203 | 0.680 to 0.748 |
| SSS-8 | 0.729 | 0.0202 | 0.694 to 0.762 |
| SSD-12 | 0.836 | 0.0158 | 0.806 to 0.863 |
| WI-8 | 0.814 | 0.0172 | 0.783 to 0.842 |
| PHQ-15 and SSD-12 combination | 0.838 | 0.0156 | 0.809 to 0.865 |
| PHQ-15 and WI-8 combination | 0.818 | 0.0171 | 0.787 to 0.846 |
| SSS-8 and SSD-12 combination | 0.836 | 0.0159 | 0.806 to 0.863 |
| SSS-8 and WI-8 combination | 0.816 | 0.0173 | 0.785 to 0.844 |

^a^ DeLong et al., 1988

^b^ Binomial exact

**Table S3. Pairwise comparison of ROC curves of PHQ-15, SSS-8, SSD-12 and WI-8 versus each other, and combination of PHQ-15 or SSS-8 with SSD-12 or WI-8 verus SSD-12 or WI-8 alone**

|  | Difference between areas | SE ^a^ | 95% CI | z statistic | p |
| --- | --- | --- | --- | --- | --- |
| PHQ-15 verus SSS-8 | 0.0139 | 0.0163 | -0.0181 to 0.0458 | 0.849 | 0.3957 |
| PHQ-15 versus SSD-12 | 0.121 | 0.0205 | 0.0810 to 0.161 | 5.908 | < 0.001 |
| PHQ-15 versus WI-8 | 0.0986 | 0.0194 | 0.0605 to 0.137 | 5.076 | < 0.001 |
| SSS-8 versus SSD-12 | 0.107 | 0.0179 | 0.0723 to 0.142 | 6.002 | < 0.001 |
| SSS-8 versus WI-8 | 0.0847 | 0.0177 | 0.0500 to 0.119 | 4.785 | < 0.001 |
| SSD-12 versus WI-8 | 0.0226 | 0.0119 | -0.000681 to 0.0459 | 1.903 | 0.0571 |
| SSD12 verus  PHQ-15 and SSD-12 combination | 0.00192 | 0.00323 | -0.00441 to 0.00826 | 0.595 | 0.5518 |
| SSD12 versus  SSS-8 and SSD-12 combination | 0.000445 | 0.00210 | -0.00366 to 0.00455 | 0.212 | 0.8319 |
| WI-8 versus  PHQ-15 and WI-8 combination | 0.00452 | 0.00372 | -0.00278 to 0.0118 | 1.214 | 0.2249 |
| WI-8 versus  SSS-8 and WI-8 combination | 0.00223 | 0.00367 | -0.00496 to 0.00941 | 0.607 | 0.5440 |

^a^ DeLong et al., 1988

**Table S4. Sensitivity, specificity, negative predictive values, positive predictive values, efficiency of PHQ-15, SSS-8, SSD-12 (n = 694)**

| PHQ-15  Score | ⩾5 | ⩾6 | ⩾7 | **⩾8** | ⩾9 | ⩾10 | ⩾11 | ⩾12 | ⩾13 | ⩾14 | ⩾15 | ⩾16 | ⩾17 | ⩾18 | ⩾19 | ⩾20 |
| --- | --- | --- | --- | --- | --- | --- | --- | --- | --- | --- | --- | --- | --- | --- | --- | --- |
| Sensitivity | 0.93 | 0.89 | 0.85 | 0.80 | 0.72 | 0.64 | 0.57 | 0.49 | 0.42 | 0.35 | 0.31 | 0.27 | 0.19 | 0.16 | 0.11 | 0.09 |
| Specificity | 0.25 | 0.33 | 0.40 | 0.52 | 0.59 | 0.66 | 0.74 | 0.80 | 0.84 | 0.88 | 0.91 | 0.93 | 0.94 | 0.96 | 0.97 | 0.98 |
| NPV | 0.39 | 0.40 | 0.42 | 0.46 | 0.47 | 0.49 | 0.53 | 0.56 | 0.58 | 0.60 | 0.63 | 0.67 | 0.63 | 0.67 | 0.64 | 0.68 |
| PPV | 0.87 | 0.86 | 0.84 | 0.83 | 0.80 | 0.78 | 0.77 | 0.75 | 0.74 | 0.73 | 0.72 | 0.71 | 0.69 | 0.69 | 0.68 | 0.68 |
| Efficiency | 0.48 | 0.52 | 0.55 | 0.61 | 0.63 | 0.65 | 0.68 | 0.69 | 0.70 | 0.70 | 0.71 | 0.70 | 0.69 | 0.69 | 0.68 | 0.68 |
| SSS-8  Score | ⩾5 | ⩾6 | ⩾7 | ⩾8 | ⩾9 | ⩾10 | ⩾11 | ⩾12 | ⩾13 | ⩾14 | ⩾15 | ⩾16 | ⩾17 | ⩾18 | ⩾19 | ⩾20 |
| Sensitivity | 0.88 | 0.85 | 0.77 | 0.72 | 0.67 | 0.6 | 0.52 | 0.47 | 0.43 | 0.39 | 0.36 | 0.30 | 0.26 | 0.21 | 0.18 | 0.14 |
| Specificity | 0.37 | 0.46 | 0.54 | 0.63 | 0.68 | 0.72 | 0.78 | 0.81 | 0.85 | 0.89 | 0.92 | 0.93 | 0.93 | 0.96 | 0.97 | 0.98 |
| NPV | 0.42 | 0.44 | 0.46 | 0.50 | 0.52 | 0.53 | 0.55 | 0.56 | 0.60 | 0.65 | 0.68 | 0.67 | 0.70 | 0.71 | 0.77 | 0.74 |
| PPV | 0.85 | 0.85 | 0.82 | 0.81 | 0.80 | 0.78 | 0.76 | 0.75 | 0.75 | 0.74 | 0.74 | 0.72 | 0.71 | 0.70 | 0.70 | 0.69 |
| Efficiency | 0.54 | 0.59 | 0.62 | 0.66 | 0.68 | 0.68 | 0.69 | 0.70 | 0.71 | 0.72 | 0.73 | 0.71 | 0.71 | 0.70 | 0.70 | 0.69 |
| SSD-12  Score | ⩾10 | ⩾11 | ⩾12 | **⩾13** | ⩾14 | ⩾15 | ⩾16 | ⩾17 | ⩾18 | ⩾19 | ⩾20 | ⩾21 | ⩾22 | ⩾23 | ⩾24 | ⩾25 |
| Sensitivity | 0.86 | 0.84 | 0.81 | **0.80** | 0.79 | 0.78 | 0.76 | 0.72 | 0.69 | 0.66 | 0.63 | 0.6 | 0.57 | 0.54 | 0.50 | 0.47 |
| Specificity | 0.65 | 0.66 | 0.69 | **0.72** | 0.74 | 0.77 | 0.80 | 0.83 | 0.83 | 0.85 | 0.85 | 0.86 | 0.87 | 0.88 | 0.89 | 0.91 |
| NPV | 0.56 | 0.56 | 0.57 | **0.59** | 0.61 | 0.63 | 0.66 | 0.68 | 0.68 | 0.69 | 0.69 | 0.69 | 0.69 | 0.69 | 0.70 | 0.72 |
| PPV | 0.90 | 0.89 | 0.88 | **0.87** | 0.87 | 0.87 | 0.87 | 0.85 | 0.84 | 0.83 | 0.82 | 0.81 | 0.80 | 0.79 | 0.78 | 0.77 |
| Efficiency | 0.72 | 0.72 | 0.73 | **0.75** | 0.76 | 0.77 | 0.79 | 0.79 | 0.79 | 0.79 | 0.78 | 0.77 | 0.77 | 0.76 | 0.76 | 0.76 |
| WI-8  Score | ⩾15 | ⩾16 | **⩾17** | ⩾18 | ⩾19 | ⩾20 | ⩾21 | ⩾22 | ⩾23 | ⩾24 | ⩾25 | ⩾26 | ⩾27 | ⩾28 | ⩾29 | ⩾30 |
| Sensitivity | 0.86 | 0.83 | **0.8** | 0.75 | 0.73 | 0.68 | 0.64 | 0.60 | 0.55 | 0.50 | 0.46 | 0.42 | 0.40 | 0.36 | 0.35 | 0.31 |
| Specificity | 0.57 | 0.62 | **0.67** | 0.73 | 0.76 | 0.80 | 0.82 | 0.85 | 0.88 | 0.89 | 0.90 | 0.92 | 0.93 | 0.95 | 0.95 | 0.96 |
| NPV | 0.50 | 0.53 | **0.55** | 0.59 | 0.61 | 0.63 | 0.65 | 0.68 | 0.71 | 0.70 | 0.71 | 0.74 | 0.74 | 0.77 | 0.79 | 0.80 |
| PPV | 0.88 | 0.88 | **0.86** | 0.85 | 0.85 | 0.83 | 0.82 | 0.81 | 0.79 | 0.78 | 0.77 | 0.76 | 0.75 | 0.74 | 0.74 | 0.73 |
| Efficiency | 0.66 | 0.69 | **0.71** | 0.74 | 0.75 | 0.76 | 0.76 | 0.77 | 0.77 | 0.76 | 0.76 | 0.75 | 0.75 | 0.75 | 0.75 | 0.74 |
